# Amplification of ammonia sensing performance through gate induced carrier modulation in Cur-rGO Silk-FET

**DOI:** 10.1038/s41598-023-34617-7

**Published:** 2023-05-19

**Authors:** Avik Sett, Lisa Sarkar, Santanab Majumder, Tarun Kanti Bhattacharyya

**Affiliations:** 1 Department of Electronics and Electrical Communication Engineering, IIT Kharagpur, Kharagpur, 721302 West Bengal India; 2 School of Nanoscience and Technology, IIT Kharagpur, Kharagpur, 721302 West Bengal India

**Keywords:** Graphene, Nanoscale materials, Nanoscience and technology

## Abstract

Uncontrolled human and industrial activities lead to the increase in demand for selective gas sensors for detection of poisonous gases in our environment. Conventional resistive gas sensors suffer from predetermined sensitivity and poor selectivity among gases. This paper demonstrates curcumin reduced graphene oxide-silk field effect transistor for selective and sensitive detection of ammonia in air. The sensing layer was characterized by X-ray diffraction, FESEM and HRTEM to confirm its structural and morphological features. Raman spectroscopy, Fourier transform infrared spectroscopy and X-ray photoelectron spectroscopy was carried out to analyze the functional moieties present in the sensing layer. Curcumin reduced graphene oxide introduces sufficient hydroxyl groups in the sensing layer to provide high degree of selectivity towards ammonia vapors. The performance of the sensor device was evaluated at positive, negative and zero gate voltage. Carrier modulation in the channel through gate electrostatics revealed that the minority carriers (electrons) in p-type reduced graphene oxide plays a pivotal role in enhancement of sensitivity of the sensor device. The sensor response was enhanced to 634% for 50 ppm ammonia at 0.6 V gate voltage compared to 23.2% and 39.3% at 0 V and − 3 V respectively. The sensor exhibited faster response and recovery at 0.6 V owing to higher mobility of electrons and quick charge transfer mechanism. The sensor exhibited satisfactory humidity resistant characteristics and high stability. Hence, curcumin reduced graphene oxide-silk field effect transistor device with proper gate bias elucidates excellent ammonia detection and may be a potential candidate for future room temperature, low power, portable gas detection system.

## Introduction

Due to the rise of activities in chemical, food and automobile industries, there is a significant demand for development of hand held and battery operated^1^ gas detectors. These demands call for intensive research towards fabrication of miniaturized, room temperature and low power gas sensors. In this context, sensing layers based on metal oxide semiconductors (ZnO, TiO_2_, SnO_2_, WO_3_ etc.^2–5^) are widely explored. These sensing layers are highly attractive due to their enormous sensitivity, but their functioning requires high operating temperatures (typically in the range of 200–450 °C). The high-power budget of metal oxides limits their usage in hand held, room temperature gas detection systems. For example, SnO_2_ based Taguchi sensors (Figaro Japan) that are available commercially, utilizes 200 mW power. Hence, efforts are required to develop room temperature, low power sensors. There are several attempts in this regard^6–9^, which aim to integrate the sensors with the matured CMOS platform. Certain gases like ammonia, hydrogen sulfide have low ignition temperature and are highly flammable. Therefore, researchers have focused towards synthesis of functionalized nanomaterials that operate at room temperatures. These materials include two-dimensional nanomaterials (graphene), transition metal di-chalcogenides (MoS_2_, WS_2_), black phosphorus, metal organic frameworks, etc.

Graphene, a two-dimensional nanomaterial has found significant attention due to its enormous surface area, thermal and mechanical stability, high mobility, and flexibility^10^. Graphene is found to be highly sensitive towards different gas analytes. The two-dimensional honeycomb structure along with single layer of carbon atoms facilitate higher sensitivity towards different analytes. Pristine graphene which is free of defects, has poor adsorption energy towards different gases. Introducing defects and dopants in the graphene matrix shall enhance the adsorption energy level and facilitate better charge transfer between the target analytes and graphene matrix. Chemical exfoliation of graphene leads to graphene oxide, which on reduction produces reduced graphene oxide (RGO). Reduced graphene oxide consists of several defect sites along with various functional moieties, that introduces several active sites for adsorption of target gases. The biggest advantage of RGO is its ability to detect trace gases even at room temperature. This makes RGO an ideal candidate to be utilized in future generation room temperature, low power, portable gas sensors.

In various industrial applications, ammonia is used as a common reagent. Ammonia is a highly pungent and toxic gas, which may cause severe effects on respiratory tracts, eyes, and skin, if exposed at higher concentrations (> 350 ppm)^11,12^. In case of prolonged exposure, severe health issues are observed in human beings, including death. According to OSHA (Occupational safety and health administration), 15–28% ammonia concentration by volume in air is considered highly dangerous to health^13^. The flammable nature of ammonia demands fabrication of sensors that must operate at room temperature. Few chemo-resistive ammonia sensors based on graphene are developed^11,14–21^, however their performances are not satisfactory. RGO based sensors that are previously reported, suffer from large response and recovery, baseline drift, poor recoverability, and unsatisfactory selectivity. Earlier reports on graphene-based gas sensors lack analysis under humidity environments. In case of resistive sensors, once the sensing layer gets deposited, it cannot be further tuned or modulated. These factors boost the motivation towards development of Field effect transistor (FET) based gas sensors, where the channel can be modulated even after the sensor is fabricated. This strategy might facilitate “sensitivity enhancement” by controlling the number of effective carriers in the channel region^22^.

In the present work, a field effect transistor (FET) has been fabricated to detect ammonia vapors selectively. Curcumin reduced graphene oxide (Cur-rGO) is synthesized as the channel material and drop casted between the source and drain electrodes. Silk is used as the gate dielectric due to (i) high dielectric constant and (ii) ease of deposition. This reduces the fabrication cost and complexities. The current work demonstrates the play of gate voltage in tuning the type of charge carriers participating in the charge transfer/sensing mechanism. Even though RGO is known to be a p-type material, it is observed that application of proper gate voltage ensures electron participation for ammonia detection. During application of positive gate bias (0.6 V), the sensor exhibits enormous sensitivity of 634% at 50 ppm ammonia. When compared to zero gate bias (purely resistive-type) and negative gate bias, ~ 15 times enhancement in response is observed. The participation of electron enhances the rate of charge transfer, which is further reflected in response and recovery time analysis. The performance of these sensors under appropriate gate voltage reveals the significance of “optimization of gate electrostatics” in chemical sensors. The use of curcumin to reduce graphene oxide is also validated through Fourier transform infrared spectroscopy. The reduction through curcumin leads to complete removal of all other functional groups except hydroxyl (O–H bonds). These hydroxyl groups play a key role in determining the selectivity of the sensors towards ammonia. Cur-rGO was further characterized by FESEM, HRTEM, Raman spectroscopy and X-ray photoelectron spectroscopy to reveal its structural, morphological, and chemical properties. The Cur-rGO based silk FET exhibited good immunity against humidity and displayed good stability. Compared to the previous reports, its is evident that Cur-rGO silk FET with appropriate gate bias is highly efficient towards selective and sensitive detection of ammonia. These results prove that Cur-rGO silk FET based ammonia sensor can be used in future commercial low power, room temperature and portable gas detector systems.

## Results

**Figure 1:**
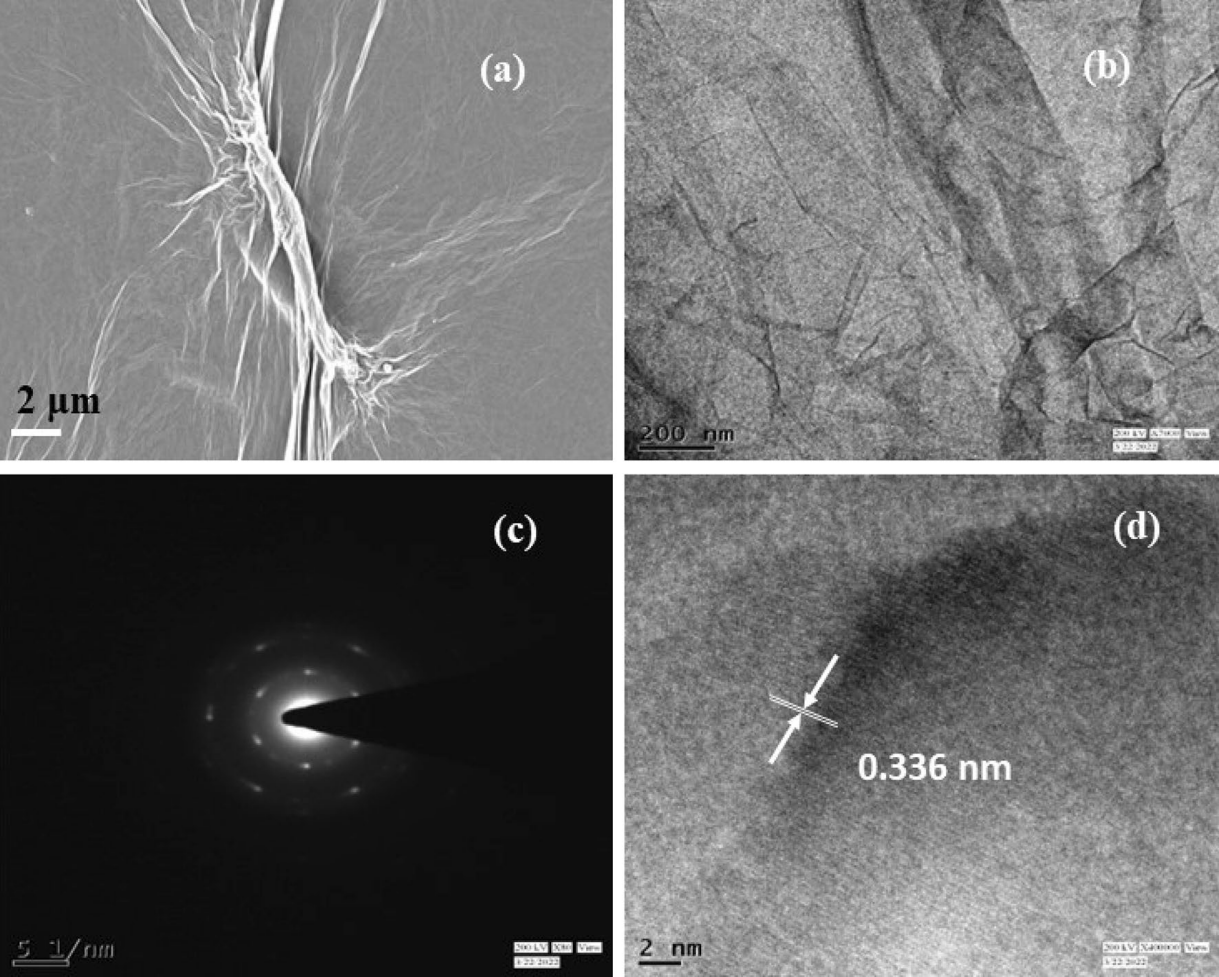
(**a**) FESEM image of Cur-rGO nanosheets, (**b**) HRTEM image of Cur-rGO nanosheet, (**c**) SAED pattern of Cur-rGO nanosheet and (**d**) lattice fringes and spacings in Cur-rGO.

Morphological, structural, and compositional analysis of Cur-rGO was carried out through FESEM, HRTEM, XRD, FTIR, XPS and Raman spectroscopy. Figure 1a depicts the FESEM image of Cur-rGO, which reveals presence of enormous wrinkles over the sheets. Presence of wrinkles and the sheet like morphology is further confirmed by HRTEM as shown in Fig. 1b. Selective area electron diffraction (SAED) pattern in Fig. 1c shows the presence of multiple rings with different spot size and variable intensity. This confirms the random orientation of the sheets and presence of numerous defects. The two diffraction planes correspond to (002) and (100) planes of Cur-rGO. Figure 1d shows the lattice fringes, revealing the value of d-spacing to be 0.336 nm, very close to pristine graphene (0.36 nm).

**Figure 2:**
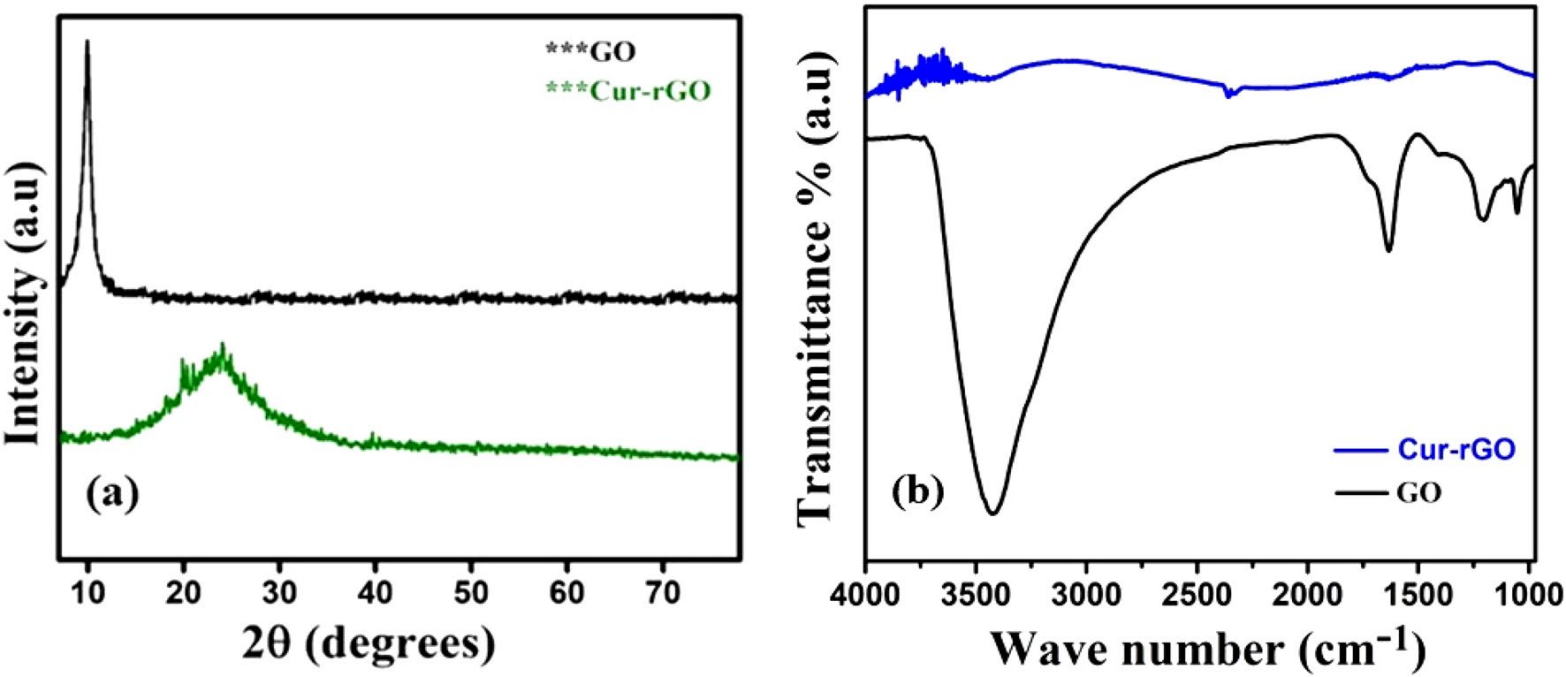
(**a**) XRD pattern of GO and Cur-rGO and (**b**) FTIR spectra of GO and Cur-rGO.

Examination of crystallographic orientation of the synthesized materials was carried out through XRD analysis having Cu Kα_1_ radiation (λ = 1.54 Å) equipped with a parallel beam diffractometer. Figure 2a shows the XRD pattern of GO and Cur-rGO. The sharp diffraction peak at 10.48° for GO, corresponds to reflection from (001) plane. This confirms the formation of graphene oxide by successful oxidation of graphite flakes. A broad peak centered at 24.57° demonstrates reflection from (002) lattice planes of Cur-rGO nanosheets. Figure 2b exhibits the FTIR analysis of GO and Cur-rGO samples. The broad peak due to O–H vibrations is reflected between 3000 and 3500 cm^−1^. The C=O bond at 1740 cm^−1^ relates to the carboxylic groups present at the edge of graphene sheet. The presence of C–O stretch at 1048 cm^−1^, C–O–C stretch at 1220 cm^−1^, C–OH stretch at 1373 cm^−1^ reflects the different functional moieties present in GO^23^. Thermal treatment of GO by curcumin leads to successful reduction of GO as confirmed by FTIR spectra. Apart from O–H stretching (centered at 3400 cm^−1^), all other functional groups are successfully removed from GO surface. The presence of this O–H group is highly influential towards selective detection of ammonia, compared to other VOCs.

**Figure 3:**
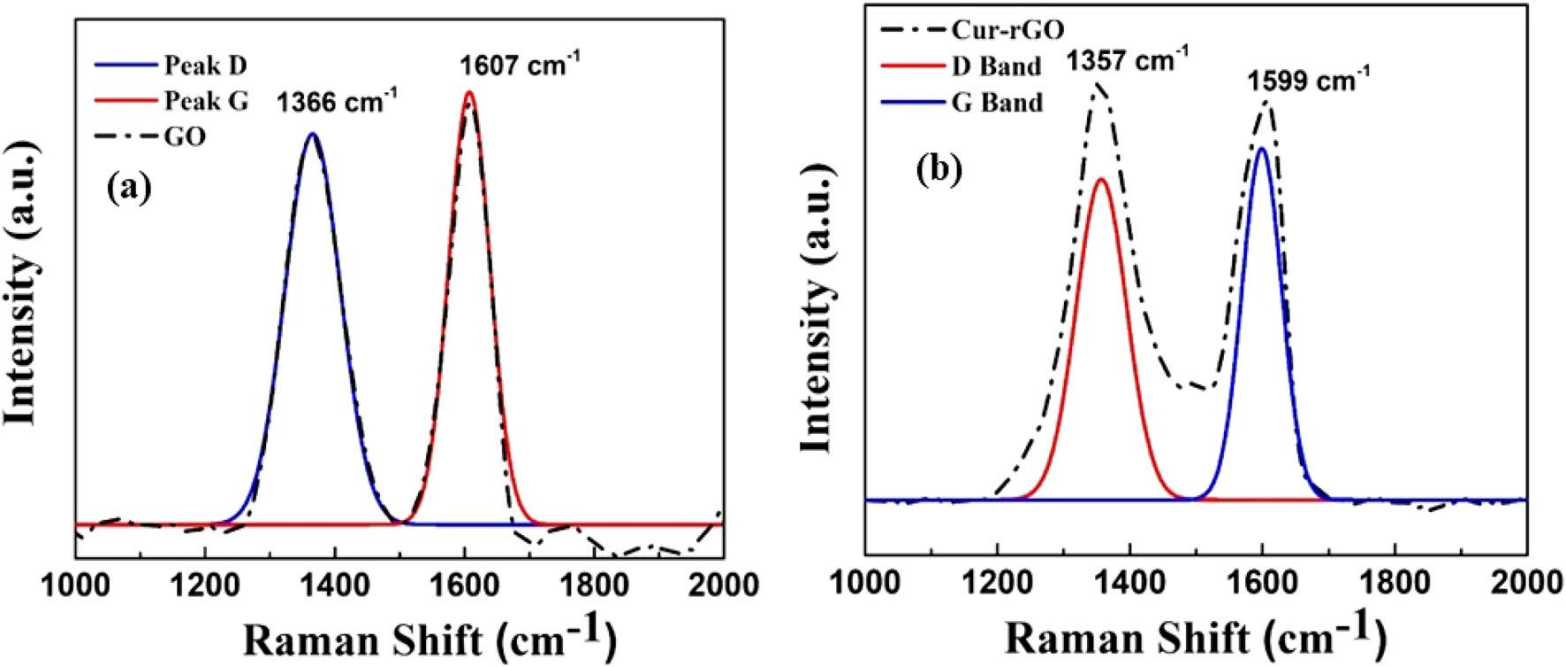
(**a**) Raman spectra for GO, (**b**) Raman spectra for Cur-rGO.

Successful reduction through curcumin is further validated by Raman spectroscopy as shown in Fig. 3. Figure 3a depicts the Raman spectra for GO whereas Fig. 3b)illustrates the Raman spectra for Cur-rGO. The D and G bands for GO was found at 1366 cm^−1^ and 1607 cm^−1^, however after reduction the D and G bands shifted to 1357 cm^−1^ and 1599 cm^−1^, which is in accordance with the previous reports^24,25^. The attachment of curcumin functionalities with the unsaturated carbon atoms leads to shifting of ‘D’ and ‘G’ bands to lower wave numbers. The electron donating curcumin leads to softening of phonons and shifts the fermi level^26,27^.

**Figure 4:**
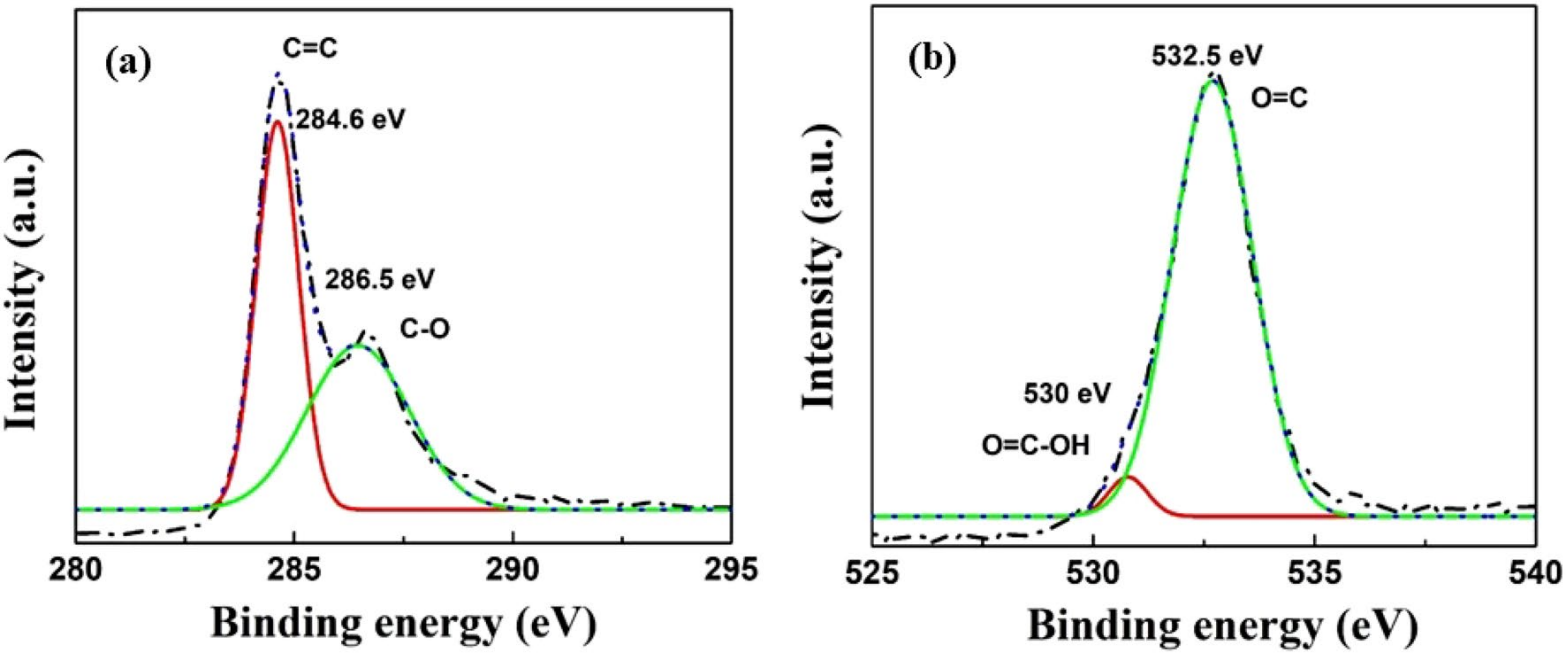
(**a**) Narrow scan spectra of C 1s and (**b**) O 1s of Cur-rGO.

The curcumin reduced GO was further investigated by X-ray photoelectron spectroscopy (XPS) to further analyze the chemical states and functional groups. The deconvoluted XPS spectrum of C 1s region of Cur-rGO is shown in Fig. 4a. It is noticed that the C 1S region exhibits the peaks with binding energies 284.6 eV and 286.5 eV corresponding to C=C and C–O bond respectively. The deconvoluted graph of O 1S region is also plotted in Fig. 4b. O 1s peaks with binding energies 530.6 eV and 532.5 eV are attributed to O=C–OH and O=C bond respectively. The presence of O=C–OH is very significant in our study due to high adsorption energy of NH_3_ molecules towards –OH groups.

## Discussions

Reduction of GO in presence of curcumin leads to the formation of a highly sensitive ammonia sensing layer. The presence of field effect in the sensor devices allows modulation of charge carriers in the channel region. As reduced graphene oxide exhibits p-type characteristics, electrons play the role of minority carriers in all transport mechanisms. These electrons play a pivotal role towards highly sensitive detection of ammonia at low concentrations. It is predicted that charge transfer during sensing would be more efficient for electrons as compared to holes. Holes with a lesser mobility and higher effective mass would face larger hindrance towards fast charge transfer. Hence, it is focused to use electron as the main carrier for charge transfer by application of appropriate gate bias.

**Figure 5:**
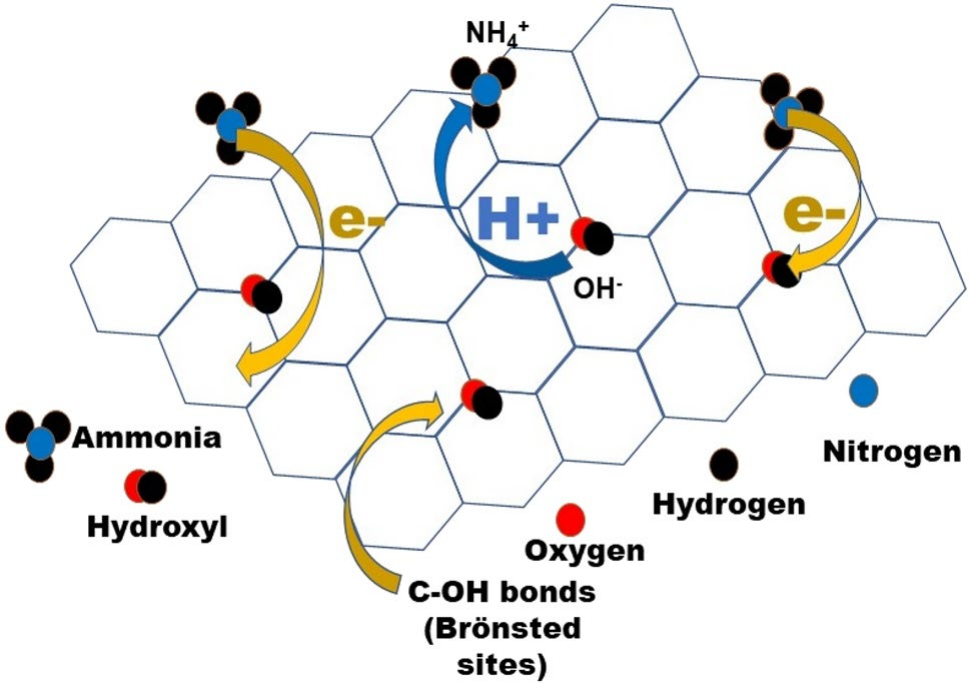
Ammonia sensing mechanism of Cur-rGO sensor.

Pristine graphene exhibits 0.114 eV adsorption energy towards ammonia^28^. The epoxy and hydroxyl groups in defect induced graphene sheets are found to have binding energies of be 0.219 eV and 0.840 eV respectively for ammonia^28^. This shows the importance of hydroxyl groups to be present over the sensor surface. When the applied gate voltage is negative or zero, holes are the carriers that take part in the sensing phenomenon. Ammonia molecules get bound to the hydroxyl groups and donates electrons to the Cur-rGO sensing layer as shown in Fig. 5. The majority carrier being holes, get recombined with the incoming electrons and the drain current reduces. When the applied voltage is positive, the channel consists of electrons. When ammonia molecules come in contact with the channel, it boosts the electron concentration of Cur-rGO and hence increases the drain current. The functionalization of the Cur-rGO surface with hydroxyl groups offer high binding energy towards ammonia leading to preferential adsorption and high selectivity. This change in drain current is visualized as the response and is expressed in percentage.

Response is mathematically defined as:

1 $$ {\text{Response }} = \, \left( {{\text{I}}_{{{\text{d}},{\text{ gas}}}} {-}{\text{ I}}_{{{\text{d}},{\text{ air}}}} } \right)/{\text{I}}_{{{\text{d}},{\text{ air}}}} *{1}00\% $$

where, I_d, gas_ and I_d, air_ are the drain current upon exposure to ammonia and air respectively.

**Figure 6:**
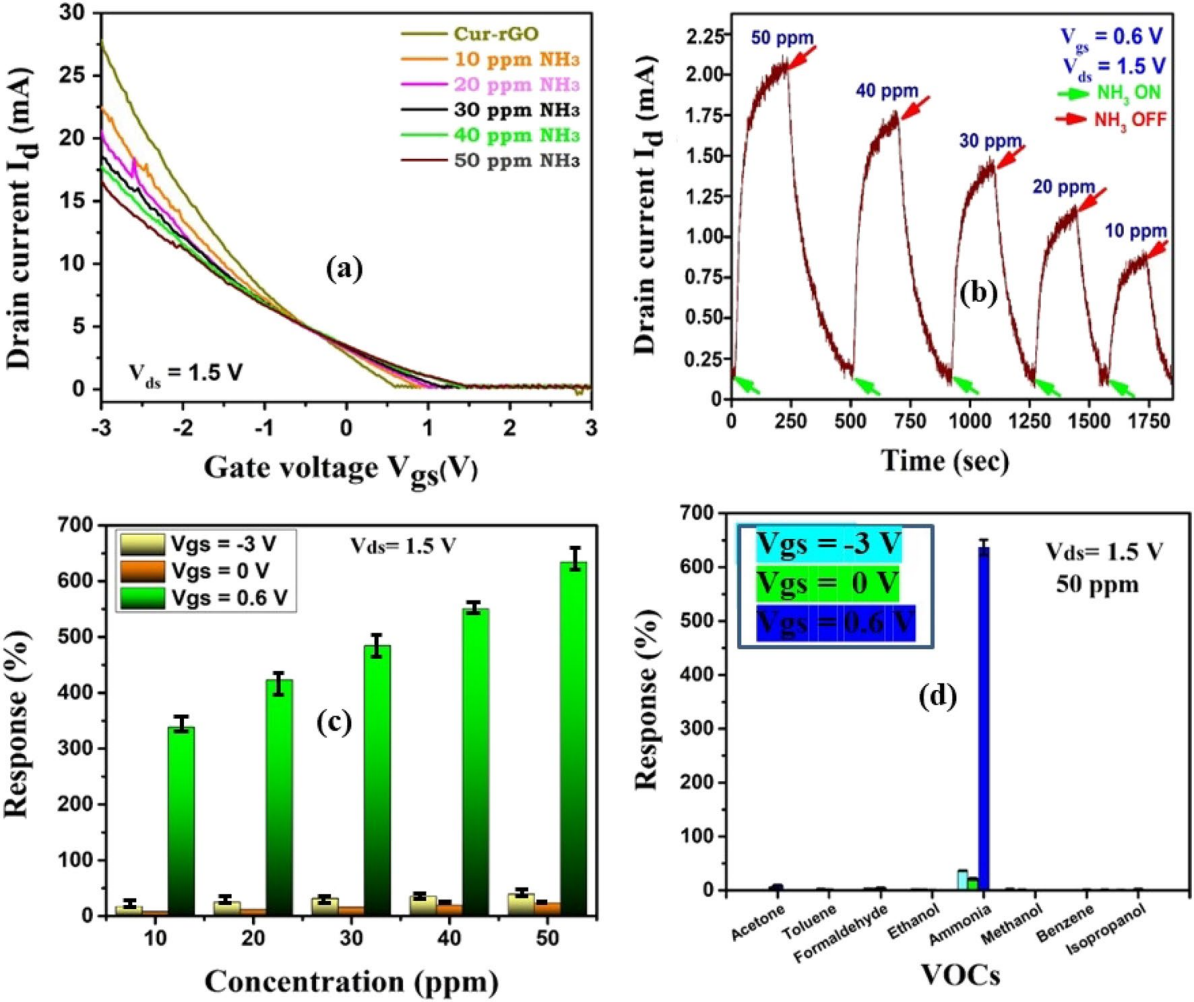
(**a**) I_d_-V_gs_ of Cur-rGO silk device along with different concentration of ammonia. (**b**) Concentration curve at V_gs_ = 0.6 V. (**c**) Response towards ammonia at different gate voltage. (**d**) Selectivity of the sensor towards ammonia.

The I_d_-V_g_ characteristics (V_ds_ = 1.5 V) in Fig. 6a shows that the device exhibits very high ON–OFF ratio (~ 10^5^). With gate voltage less than 0.5 V, the device exhibits conductivity due to holes. However, after 0.5 V, the device exhibits electron conductivity. When the sensor is exposed to ammonia molecules, the hole current reduces (left of 0.5 V) but the electron current increases (right of 0.5 V). When the majority carriers are holes, then presence of ammonia molecules reduces the number of holes due to electron–hole recombination. Hence, a reduction in current is observed. With increased gate voltage above 0.5 V, the presence of ammonia donates electron in the channel. Now, electron being the majority carrier, its concentration increases and hence increase in current is observed.

Figure 6b depicts the change in current with change in concentration of NH_3_ from 10 to 50 ppm. The concentration curve is shown at V_gs_ = 0.6 V, the best sensing condition. Figure 6c shows the response of the sensor towards ammonia at different gate voltage. It is evident that at 0 V gate, the response is very low. At negative voltage, with holes as the majority carrier, the sensitivity increases, however not by much. At positive gate voltage, specifically at 0.6 V, the electrons are the primary carriers that take part in sensing. At that voltage, two events enhance the sensing phenomenon: (i) a smaller number of electrons in the channel, facilitating huge response in presence of trace amount of ammonia and (ii) mobility of electron being higher than holes, facilitates faster charge transfer. At 50 ppm concentration, the response towards ammonia is found to be 23.2% at 0 V V_gs_ and 39.3% with − 3 V V_gs_. However, at V_gs_ = 0.6 V, there is tremendous increase in response to 634% for 50 ppm ammonia. This result signifies the essence of gate voltage optimization to increase the sensitivity of the sensing layer.

**Figure 7:**
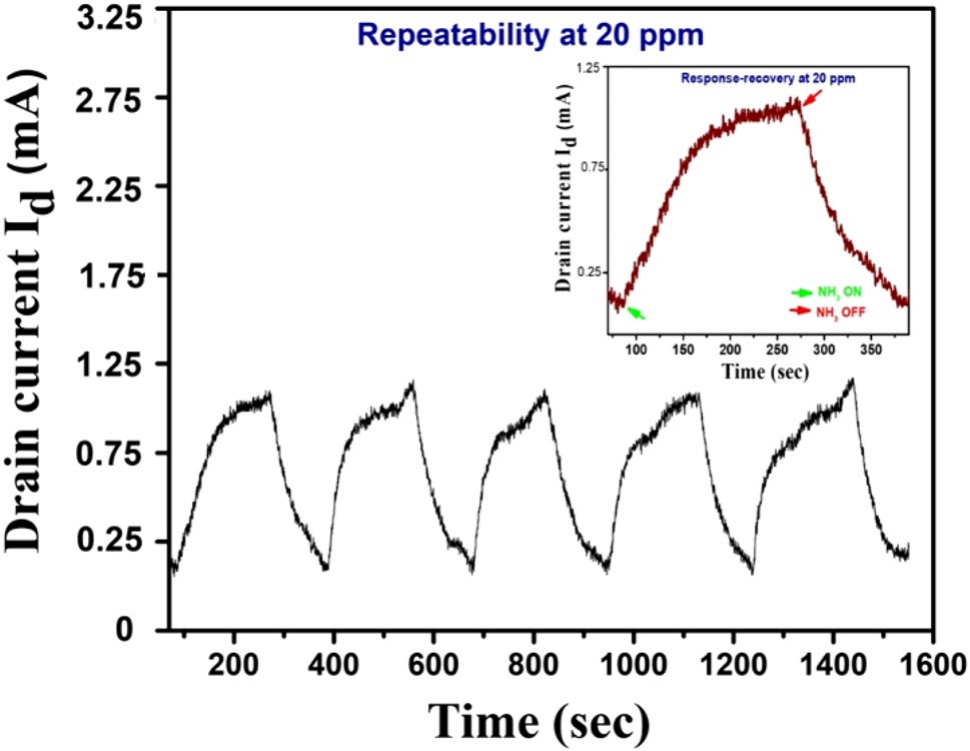
Repeatability of the device at 20 ppm.

Figure 6d shows the high degree of selectivity of Cur-rGO towards NH_3_ molecules when compared to seven other VOCs (acetone, toluene, formaldehyde, ethanol, methanol, benzene, isopropanol). The current is observed to decrease with decrease in concentration, due to the n-type behavior of the channel at the desired gate voltage. Figure 7 depicts the repeatability of Cur-rGO towards 20 ppm NH_3_ at an optimized V_gs_ of 0.6 V. The sensor reaches the baseline current after the gas source is removed. The absence of baseline drift is a blessing in terms of calibration of the device.

**Figure 8:**
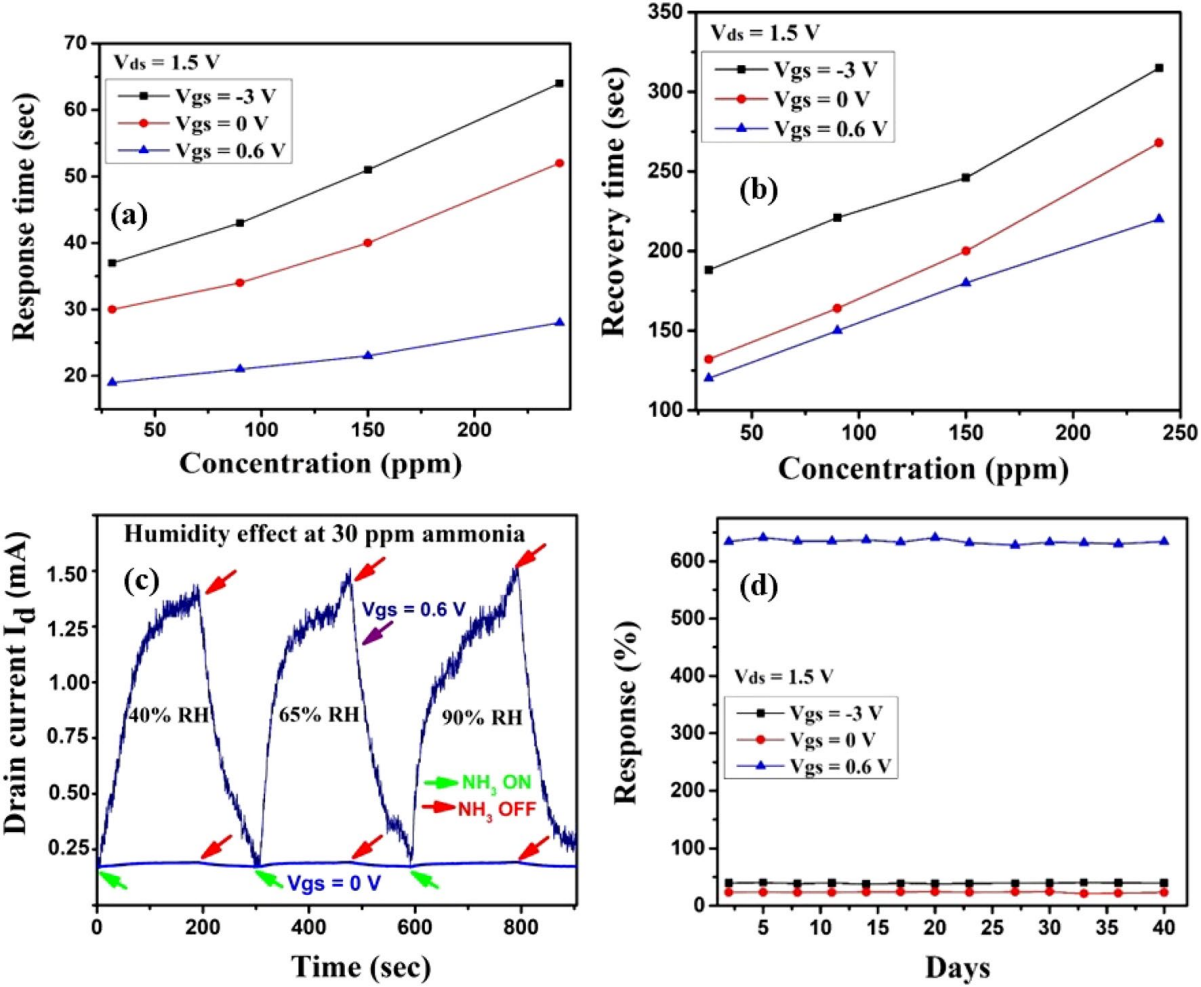
(**a**) Response time variation of Cur-rGO with gate voltage. (**b**) Recovery time variation of Cur-rGO with gate voltage. (**c**) Humidity resistant behaviour of Cur-rGO. (**d**) stability of the device for 40 days.

Response time is the time taken by the sensor response to reach 90% from 10% of the maximum value. Recovery time is the time taken by the sensor to recover from 90 to 10% of the maximum value. The response and recovery time of Cur-rGO at positive gate voltage (0.6 V) is lower compared to negative (− 3 V) and zero gate bias as shown in Fig. 8a,b. This is since electron mobility is higher compared to holes and an optimized number of electrons in the channel boosts the sensitivity of Cur-rGO. The humidity resistant nature of Cur-rGO Silk-FET device is very significant for developing highly accurate ammonia detectors. The variation of response with RH% at V_gs_ = 0.6 V and V_gs_ = 0 V is illustrated in Fig. 8c. It is observed that the sensor is immune to humidity variations. This can be attributed to the selective removal of other oxygen containing groups (carbonyl, epoxy, carboxylic) which otherwise occurs in graphene oxide. Stability of the sensor device is elucidated in Fig. 8d, where it is clearly observed that Cur-rGO Silk-FET is highly stable even after 40 days.

Hence, the effect of gate voltage towards enhancement of ammonia sensing performance for Cur-rGO introduces a new technique towards increasing of sensitivity in gas sensing domain. The performance of Cur-rGO towards NH_3_ is compared with previous works from literature as depicted in Table 1. It is thus observed that Cur-rGO Silk-FET proves to be highly efficient towards detection of NH_3_ at room temperature. The optimization of gate voltage to change the polarity of the channel and the presence of silk as dielectric ensures sensitive as well as low power operation. The proper reduction of graphene oxide by curcumin leaves behind hydroxyl groups, which facilitates selective ammonia sensing behaviour of the Cur-rGO Silk FET. Therefore, Cur-rGO Silk FET can be used in future commercial room temperature, low power ammonia gas detection systems.

**Table 1:** Comparison between different Ammonia sensors reported in literature.

Sensor type/operating temperature	Response (in %)	Concentration (ppm)	Response and recovery time	Ref
RGO	930	400	31 s, 500 s	^18^
RGO	80	10	53 s, 554 s	^19^
RGO	12	800	505 s, 1340 s	^20^
In_2_O_3_ (300 °C)	700	800	12 s, 18 s	^30^
ZnO (100 °C)	180	800	48 s, 10 s	^30^
SnO_2_ (100 °C)	190	800	36 s, 25 s	^30^
In_2_O_3_/RGO (25 °C)	110	15	17 s, 214 s	^30^
Cur-rGO (25 °C) at V_gs_ 0 V	23.2	50	41 s, 198 s	Present work
Cur-rGO (25 °C) at V_gs_ − 3 V	39.3	50	30 s, 140 s	Present work
Cur-rGO (25 °C) at V_gs_ 0.6 V	634	50	18 s, 122 s	Present work

## Methods

Graphite powder, sodium nitrate, potassium permanganate, sulphuric acid, ammonia solution, ethanol, sodium carbonate, calcium chloride, formic acid and curcumin were obtained from Merck India pvt Ltd. and were used throughout the experiment without further purification. Commercially available Bombyx Mori silk was purchased from M/S Bombyx Mori Silks and Textiles, Srinagar, India. Millipore purification system was used for preparing de-ionized water.

Widely known Hummer’s method was implemented towards synthesis of graphene oxide (GO) sheets by chemical exfoliation of graphite powder. For synthesis of curcumin reduced graphene oxide (Cur-rGO), solution of curcumin in ethanol was prepared by adding 5 mg curcumin to 10 ml ethanol. The solution was stirred for an hour to obtain a homogenous yellow solution. A beaker containing 100 ml GO dispersion (1 mg/ml) was constantly stirred and curcumin solution was added in dropwise manner to obtain a homogenous mixture. After stirring for 2 h, 20 μL of ammonia solution (27%) was added to attain a pH of 10. After further stirring for an hour, the solution was transferred to a blue capped reagent bottle. The solution was then heated at 85 °C in a hot air oven for 4 h. The resulting solution was cooled to room temperature and subjected to 2500 rpm centrifugation to eliminate unwanted impurities. The as prepared solution was termed Cur-rGO.

Dielectric silk solution was prepared by a method proposed by Sarkar et al.^29^. Initially, a glue-like substance (Sericin), which binds the silk fibroins was removed. The fibroins were then dissolved to form a homogeneous solution. Sericin was removed from silk fibroins by treating it with 0.5% sodium carbonate-water solution at 110 °C for 1.5 h. In the next step, it was rinsed thoroughly with DI water and dried overnight. After the drying stage, the degummed silk was dissolved in a solution comprising of formic acid and calcium chloride (19:1), leading to regenerated silk fibroin solution. The presence of inorganic salts with formic acid facilitates dissolution of degummed silk. Calcium chloride penetrates the structure while formic acid enables swelling of silk. Penetration of these salts disrupts the hydrogen bonding between the fibroin protein chains and decomposes it to micro-sized fibrils and finally to nanofibrils. The solution was then casted onto a petri dish and dried at 60 °C. After drying, the film was immersed overnight in DI water to remove calcium chloride, as it causes detrimental effects on thin film properties. The film can be dissolved and reconstituted in formic acid directly, as, and when required.

**Figure 9:**
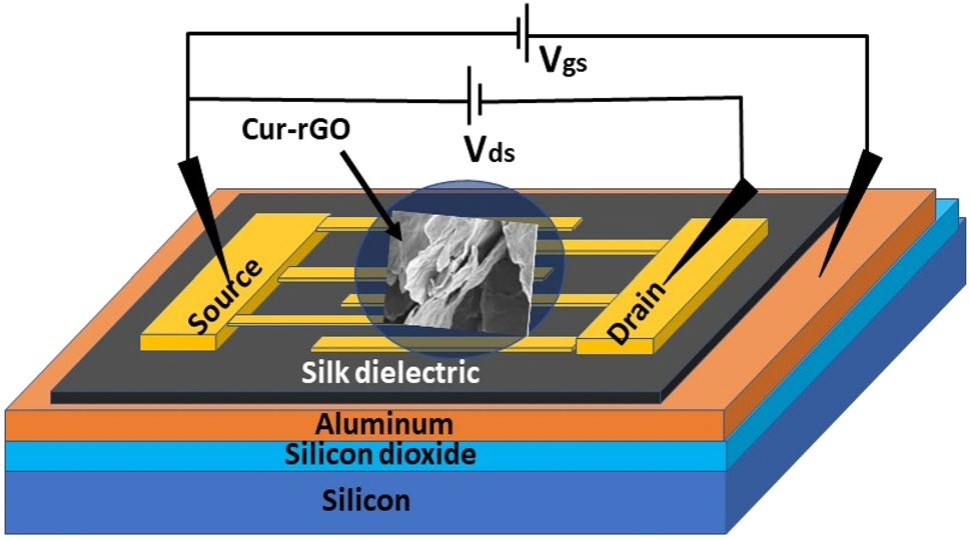
Schematic of Silk-FET sensor device with Cur-rGO as channel material.

Fabrication of silk gate field effect transistor (Silk-FET) was carried out on p <100> type silicon wafer having resistivity 4–20 Ω-cm. A 200 nm thermal oxide (dry–wet-dry) was grown over silicon to achieve proper insulation. Deposition of 50 nm aluminum layer was carried out by thermal evaporation, which acted as the gate electrode. Dielectric layer of 200 nm silk solution was spin coated over the aluminum film. Source and drain electrodes were fabricated using DC sputtering, 20 nm titanium (Ti) adhesive layer was deposited followed by 100 nm gold (Au) through a shadow mask. Interdigit gap of 200 μm was achieved between the fingers. The width of the electrodes was 500 μm. The sensing material (Cur-rGO) was deposited onto the gaps by simple drop casting technique. The schematic of the fabricated Silk-FET device is depicted in Fig. 9.

## Data Availability

The datasets used and/or analyzed during the current study are available from the corresponding author on reasonable request.
